# Factors associated with involuntary admissions among patients with substance use disorders and comorbidity: a cross-sectional study

**DOI:** 10.1186/1472-6963-13-57

**Published:** 2013-02-12

**Authors:** Anne Opsal, Øistein Kristensen, Tor K Larsen, Gro Syversen, Bakke Elise Aasen Rudshaug, Arne Gerdner, Thomas Clausen

**Affiliations:** 1 Sørlandet Hospital, Addiction Unit, Postbox 416, Kristiansand N-4604, Norway; 2 Regional Centre for Clinical Research in Psychosis, Stavanger University Hospital, Psychiatric Division, Stavanger, Norway; 3 Adult Addiction Treatment Unit, Centre for Addiction, Oslo University Hospital, Oslo, Norway; 4 School of Health Sciences, Jönköping University, Jönköping, Sweden; 5 Centre for Addiction Research (SERAF), Institute of Clinical medicine, University of Oslo, Oslo, Norway

**Keywords:** Substance use disorder, Comorbidity, Involuntary admission, Mental disorders

## Abstract

**Background:**

To investigate factors associated with involuntary admissions to hospital pursuant to a social services act of patients with substance use disorder by comparing the socio-demographic characteristics, substance use, and psychiatric comorbidities with voluntarily admitted patients.

**Methods:**

This cross-sectional study compared two groups admitted to combined substance use disorder and psychiatry wards. Sixty-five patients were involuntarily admitted pursuant to the Social Services Act and 137 were voluntarily admitted. The International Classification of Diseases and Related Health Problems was used for diagnostic purposes regarding substance use disorders, type and severity of psychiatric problems, and level of functioning. Socio-demographic variables were measured using the European Addiction Severity Index, and the Symptom Checklist-90-R instruments were used to evaluate the range of psychological problems and psychopathological symptoms. Logistic regression was performed to investigate the relationship between involuntary admissions and patients characteristics.

**Results:**

Patients who had been involuntarily admitted were more likely to be females, had utilized public welfare services more often, presented more severe substance use patterns, and had a history of more frequent visits to physicians for somatic complaints in the last 6 months, they also had fewer comorbid mental disorders. Still, considerable burdens of comorbid substance use disorders and mental disorders were observed both among involuntary and voluntary admitted patients.

**Conclusions:**

More attention is required for involuntarily admitted patients in order to meet the needs associated with complex and mixed disorders. In addition, treatment centers should offer diagnostic options and therapy regarding substance use, psychiatric and somatic disorders.

## Background

Substance dependence is a chronic relapsing disease that typically leads to psychiatric, somatic, and social comorbidities, often with shortened life expectancy [1]. In contrast to other patients with chronic diseases, patients with substance use disorders (SUDs) sometimes refuse treatment owing to denial of their own disorder, feelings of hopelessness, or a negative attitude toward treatment [2,3]. In many countries, the involuntary admission of SUD patients to institutions has been a controversial option when voluntary care has proven unsuccessful [4]. As of 2001, 73 of 90 countries worldwide provided some form of compulsory commitment (acute or rehabilitative) motivated by the intent to protect an otherwise legally capable individual who is in a self-destructive and vulnerable situation because of substance use [5]. In the literature, three main legislative domains have been described as foundations for the mandated treatment of SUD patients: mental health care acts or social services acts (in combination called civil commitment), and criminal justice acts. Although most countries may apply one or more of these acts to SUD patients, not all countries provide all three alternatives [4]. Literature from the United States has been dominated by drug courts’ studies of treatment as an alternative to prison [6]. Psychiatric research often reports on involuntary versus voluntary treatment. Less focus has been paid to SUD patients in treatment pursuant to the mental health care acts, although a considerable proportion of these patients are SUD patients [7,8]. Comorbid disorders are pervasive in mental health and SUD treatment settings, and there is a need for health service providers both in Norway and in other countries to develop better integrated services that addresses both problem areas [9].

The Norwegian Mental Health Care Act (§§ 3.2 and 3.3) is designed for patients based on their need for psychiatric care. Involuntary admission is an option only for persons who are found by a medical professional or psychologist to be incapable of assessing their own need for care. Such incapacity is only stated when patients are severely mentally ill (psychotic) and may be a danger to themselves or others [10]. In 2010, there were approximately 8300 involuntary admissions to mental health care units in Norwegian hospitals pursuant to this law [11]. Approximately one-third of patients involuntarily admitted to mental health care hospitals had a substance use disorder [12].

The Norwegian Social Services Act (§ 6.2) warrants involuntary interventions for non-psychotic adult patients with SUDs. The act covers an option for retention (up to three months) when the health of the patient is seriously at risk because of extensive and prolonged substance use, and voluntary efforts have shown to be insufficient. In Norway, voluntarily and involuntarily admitted SUD patients are often treated within the same wards, and using the same kind of therapy. In the acute phase, the main target of retention is to provide life-saving treatment; in the longer term, the aim is to motivate patients to enter voluntary treatment [13]. During 2010, only 106 decisions were made for the involuntary admission of SUD patients to institutions pursuant to the Social Services Act [14].

Involuntary commitment of non-psychotic SUD patients, although a relatively marginal phenomenon, is controversial. To force someone to treatment has many ethical considerations and can also be a traumatic experience that may result in a crisis. Worldwide, there is growing concern regarding ethical issues related to the use of involuntary treatment. Such interference with personal autonomy should not be applied without an evidence-based foundation [15]. In Sweden, the phenomenon of SUD patients in compulsory care pursuant to social services acts has to some extend been explored [16]. However, the acts in these two countries differ considerably and Swedish results may not be directly applicable to Norwegian settings. Thus far, little is known about the characteristics of involuntarily admitted patients in Norway and the factors associated with these admissions. This study addresses this knowledge gap and focuses on SUD patients who have been involuntarily admitted to institutions.

### Aims of the study

The aims of the study were to investigate factors associated with involuntary admission to treatment institutions by describing the socio-demographic characteristics, substance use patterns, and psychiatric comorbidities among SUD-patients involuntarily admitted to hospital pursuant to the Social Services Act by comparing them with voluntarily admitted patients.

## Methods

### Study subjects

This cross-sectional study compared two groups: involuntarily and voluntarily admitted patients to substance use disorder and psychiatry wards. Involuntarily admitted (IA) patients were included from three different publicly funded treatment centers in the southeastern part of Norway. The centers were located in Kristiansand, Tønsberg, and Oslo, and had 4, 4, and 3 beds for IA patients, respectively. All of the voluntarily admitted (VA) patients were from the same ward of the Kristiansand center as the IA patients. All wards were multidisciplinary (psychiatrists, psychologists, social workers, occupational therapists, specialized nurses, and other trained staff) and had specialized units that offered treatment for patients with primary SUD often combined with mental disorders (except psychosis). Treatment included assessments of somatic and mental health, with diagnoses based on a structured interview and examination in accordance with the International Classification of Diseases and Related Health Problems, 10th Revision (ICD-10); pharmacotherapy; cognitive milieu therapy; and individual motivation enhancement. The patient population was recruited mainly from urban and suburban areas.

Recruitment for the study continued consecutively from January 1, 2009 to May 31, 2011. The criteria for inclusion were as follows: substance abuse or dependence, age ≥ 18 years, understanding/speaking the Norwegian language and at least 3 weeks admission.

Before inclusion, both the IA group and the VA group of patients were detoxified, verified by either negative urine tests of alcohol, opioids, central stimulants (amphetamines, methamphetamines, and cocaine), benzodiazepines, and cannabis, or a minimum of 14 days spent in detoxification to establish baseline values not influenced by withdrawal symptoms. Patients with mental retardation (IQ < 70) who were not able to understand the questionnaires were excluded. Because pregnant SUD patients are treated in special wards, they were not included in this study.

Altogether, 103 consecutive IA patients were identified. Fifteen did not meet the inclusion criteria (12 because their stay was too short, and 3 because of insufficient mental capacity), 11 were not asked to participate owing to logistical issues. Of the 77 patients eligible for inclusion 12 refused to participate. Therefore, the rate of consent to participate was 84% (65 patients). There were 223 VA patients identified; 72 patients were excluded (69 because their stay was too short, 3 because of insufficient mental capacity). Of the remaining 151 VA patients, 14 refused to participate. Therefore, rate of consent in the VA group was 91% (137 patients).

The study was approved by The Regional Committee for Research Ethics in Norway (REK 08/206d, 2008/2900, 09/2413) and by the Privacy Issues Unit, Norwegian Social Science Data Services (NSD no. 18782). Written informed consent was obtained from all study participants.

### Instruments and measures

The ICD-10 was used for diagnostic purposes regarding current substance use disorders, current type and severity of psychiatric problems, and level of functioning [17]. All patients were interviewed according to a clinical psychiatric examination supported by the Mini-International Neuropsychiatric Interview (MINI) version 2005. The MINI is a short psychiatric interview for the assessment of psychiatric disorders in accordance with the Diagnostic and Statistical Manual of Mental Disorders, Fourth Edition (DSM-IV) and ICD-10 classification systems [18], and has high acceptance and validity [19,20]. The interviews were conducted by senior psychiatrists and psychologists who had many years of clinical and research experience with the psychiatric assessment of patients with physical disorders. In the statistical analysis, psychiatric diagnoses were categorized as serious mental illness (F 20–39, which includes schizophrenia and mood disorders) or other mental illnesses (F 40–99) [21]. Injecting illicit drugs during the past 6 months before admission and lifetime prevalence of overdoses were used as indicators for severe substance use disorder.

Socio-demographic variables were measured using the European Addiction Severity Index (EuropASI); a personal, structured interview designed for both clinical and research purposes. It includes 7 areas: medical status, employment and support status, drug and alcohol use, legal status, family history, family and social relationships, and psychiatric status [22]. The EuropASI interviews were performed by trained and certified staff. Specific substance use patterns based on the EuropASI were dichotomized into drug consumption at least once weekly versus less than weekly. The Symptom Checklist-90-R (SCL-90-R) instrument was used to evaluate the range of psychological problems and symptoms of psychopathology. The SCL-90-R test contains 90 items, measures 9 primary symptom dimensions, and provides an overview of a patient’s symptoms and their intensity. Each of the 90 items is rated on a five-point Likert-type scale, ranging from “not at all” (0) to “extremely” (4): higher values indicate greater symptom severity during the past week. The Global Symptom Index (GSI) score was used to assess the level of general psychological distress [23].

### Analysis and statistical methods

Continuous variables are reported with means and standard deviations. Categorical variables are reported as frequencies. The independent sample *t*-test, Chi-squared test, and Fisher’s exact test were used to test for statistically significant differences between groups. Logistic regression was performed to investigate the relationship between involuntary admissions and patient characteristics. Results are presented with 95% confidence intervals. Continuous variables were checked for correlation with Spearman’s rho. None of the included continuous variables had a correlation exceeding 0.7. The number of cases in the sample restricted the predictors included in the model. From univariate analysis, variables with a *P*-value < 0.10 were included in the multivariate analyses except “overdoses on drugs” because of multicollinearity with the variable “injecting illicit drugs”. The threshold for statistical significance was *P* < 0.05. Analyses were performed using SPSS 18.0 Software (SPSS Inc., Chicago, IL, USA).

## Results

### Patient characteristics

We found several differences between the IA and the VA groups (Table 1). There were significantly more female patients among the IA patients compared with the VA patients (48% vs. 27%; *P* = 0.004). During the 6 months prior to admission, significantly more IA patients received financial support from public welfare benefits, and were more often injecting illicit drugs. In addition, IA patients had experienced more overdoses during their lifetime compare with VA patients. IA patients had also significantly more frequent visits to physicians for somatic complaints during the 6 months prior to admission. However, the burden of psychological symptoms (SCL-90-R and suicide attempts) was higher in the VA group.

**Table 1:** Baseline socio-demographic variables and mental stress scores for substance abuse patients voluntarily or involuntarily admitted to addiction treatment centers

	**Involuntary *n*/ Voluntary *n***	**Involuntary**	**Voluntary**	***P*-value**
Age, mean (SD)	65/137	29 (10.6)	31 (8.9)	0.229
Females (%)	65/137	31 (47.7)	37 (27)	0.004
*Education*				
Mean years in primary and high school (SD)	59/130	10.53 (1.4)	10.59 (1.6)	0.783
Mean years in college/university (SD)	59/130	0.17 (0.8)	0.31 (1.0)	0.352
*Sources of financial support*^*a, c*^				
Employment (%)	60/130	6 (10.0)	24 (18.5)	0.137
Unemployment compensation (%)	60/130	2 (3.3)	4 (3.1)	
Public welfare benefits (%)	62/135	59 (95.2)	115 (85.2)	0.043
Partner, family, or friends (%)	60/130	17 (28.3)	37 (28.5)	0.985
Illegal activity (%)	60/130	24 (40.0)	47 (36.2)	0.610
Prostitution (%)	60/130	3 (5.0)	1 (0.8)	
*Living arrangement*^*c*^				
With partner (%)	60/130	8 (13.3)	11 (8.5)	0.298
Alone (%)	60/130	31 (51.7)	62 (47.7)	0.610
With family (%)	60/130	9 (15.0)	26 (20.0)	0.409
Homeless (%)	60/130	9 (15.0)	16 (12.3)	0.610
Institution (%)	60/130	2 (3.3)	15 (11.5)	
Visits to physician for somatic complaints^c^ (%)	60/130	24 (40.0)	32 (24.6)	0.031
Injecting illicit drug^c^ (%)	61/134	43 (70.5)	62 (46.3)	0.002
Drug overdoses^d^ (%)	59/130	41 (69.5)	63 (48.5)	0.007
Suicide attempts^d^ (%)	60/131	23 (38.3)	71 (54.2)	0.042
*Mental stress score*				
SCL-90-R GSI^b^, mean (SD)	62/135	1.04 (0.7)	1.28 (0.7)	0.023

^a^Some have more than one source of financial support.

^b^SCL-90-R GSI, Symptom Check List-90-revised, Global Symptom Index.

^c^Last 6 months before admission, ^d^Lifetime prevalence.

### Mental health diagnoses and substance use disorders

All patients met the ICD-10 criteria for current substance dependence or abuse. IA patients were using alcohol, benzodiazepines and other sedatives, and heroin significantly more often than VA patients (Table 2). Significantly more IA patients received “no mental diagnoses”, but among those with comorbid mental disorders, no significant differences were observed between the two groups regarding mental health diagnosis. The most common mental diagnoses among both IA and VA patients were F40-49 neurotic disorders, F 90 attention-deficit hyperactivity disorder (AD/HD), and F30-39 mood disorders. Among the personality disorders (F 60), emotionally unstable personality disorder (F 60.3) was the most common in both groups (8% of IA patients and 7% of VA patients) (Table 3).

**Table 2:** Substance use disorders with respect to ICD-10 diagnosis and Addiction Severity Index (ASI) of patients voluntarily and involuntarily admitted to addiction treatment centers

	**Involuntary *n*/ Voluntary *n***	**Involuntary (%)**	**Voluntary (%)**	***P-value***
*F10-19 Substance use disorders*				
Single substance	65/137	9 (13.8)	32 (23.4)	0.116
Two substances	65/137	11 (16.9)	30 (21.9)	0.412
Three or more substances	65/137	45 (69.2)	75 (54.7)	0.050
*ASI, Substance Abuse*^*ab*^				
Alcohol	60/132	29 (48.3)	41 (31.1)	0.021
Heroin	61/134	21 (34.4)	18 (13.4)	0.001
Other opiates	60/130	11 (18.3)	25 (19.2)	0.883
Benzodiazepines, other sedatives	60/134	39 (65.0)	63 (47.0)	0.020
Amphetamines	62/135	35 (56.5)	67 (49.6)	0.374
Cannabis	61/133	32 (52.5)	71 (53.4)	0.905
Cocaine, inhalants, hallucinogens	60/132	12 (20.0)	18 (13.6)	0.260

^a^Some patients abuse more than one substance, ^b^Last 6 months before admission.

**Table 3:** Current ICD-10 diagnoses of patients voluntarily and involuntarily admitted to addiction treatment centers

**Diagnosis**	**Involuntary (%)**	**Voluntary (%)**	***P-value***
*Mental disorders*			
No mental diagnosis	26 (40.0)	35 (25.5)	0.037
Severe mental diagnoses (F20-F39)	14 (21.5)	38 (27.7)	0.347
Other mental diagnoses (F40-F99)	25 (38.5)	64 (46.7)	0.270
*F 20–90 Mental disorders*^*a*^			
(F 20.) Schizophrenia disorders	5 (7.7)	5 (3.6)	0.297
(F 30.) Mood disorders	9 (13.8)	33 (24.1)	0.094
(F 40.) Neurotic disorders	18 (27.7)	48 (35.0)	0.298
(F 50.) Behavioral syndromes	1 (1.5)	4 (2.9)	
(F 60.) Personality disorders	8 (12.3)	22 (16.1)	0.484
(F 70.) Mental retardation	1 (1.5)	0	
(F 80.) Developmental disorders	1 (1.5)	0	
(F 90.) Attention-deficit hyperactivity disorders	11 (16.9)	35 (25.5)	0.172
*N=*	65	137	

^a^The number of diagnoses is higher than the number of patients because some patients have more than one mental diagnosis.

Multiple logistic regression analysis was used to investigate whether being involuntarily admitted to an institution was associated with any specific patterns. Women, receiving public welfare benefits, and more frequent visits to physicians for somatic complains, or injection of drugs during 6 months prior to treatment were associated with involuntarily admission pursuant to the Social Services Act (Table 4). Neither the severity of the mental illness nor the number of substance use diagnoses were associated with being involuntarily admitted.

**Table 4:** Logistic regression analysis on involuntary admission to addiction treatment centers pursuant to the Norwegian social services act of independent variables

**Characteristics**	**Bivariate analysis OR**^**a**^**(95% CI)**	***P*-value**	**Multivariate analysis OR**^**b**^**(95% CI)**	***P*-value**
Female gender	2.464 (1.331-4.561)	0.004	2.424 (1.174-5.003)	0.017
Public welfare benefits	3.420 (0.977-11.979)	0.054	4.029 (1.022-15.877)	0.046
Visits to physician for somatic complaints^c^	2.042 (1.063-3.921)	0.032	2.208 (1.032-4.725)	0.041
*Substance use disorders*				
Single substance	reference		reference	
Two substances	1.304 (0.474-3.587)	0.608	0.626 (0.184-2.129)	0.454
Three or more substances	2.133 (0.933-4.876)	0.072	1.034 (0.381-2.809)	0.947
Injection drug abuse in the last 6 months	2.774 (1.453-5.296)	0.002	2.925 (1.338-6.392)	0.007
*Mental disorders*				
No mental diagnosis	reference		reference	
Severe mental diagnoses (F20-F39)	0.496 (0.224-1.099)	0.084	0.532 (0.196-1.443)	0.215
Other mental diagnoses (F40-F99)	0.526 (0.265-1.045)	0.066	0.679 (0.293-1.547)	0.367
Suicide attempts (lifetime)	0.525 (0.282-0.980)	0.043	0.519 (0.244-1.105)	0.089
Scl-90-R GSI	0.581 (0.363-0.929)	0.023	0.693 (0.401-1.199)	0.190

^a^unadjusted OR, ^b^adjusted OR, ^c^Last 6 months before admission.

## Discussion

Overall, SUD patients in the IA group were characterized by severe drug dependence (defined as injection of drugs and high prevalence of overdoses) often combined with the need for public welfare benefits and a history of more frequent visits to physicians for somatic complaints. Many IA patients experience problems in multiple domains such as; SUD, somatic and psychiatric disorders as well as social strains. This “multiple domain feature” is better describing the IA patients’ situation rather than the sole focus on variations within one domain, such as the different drugs within SUD or the different diagnoses i.e. within psychiatric disorders. Comorbid substance use disorders and mental disorders were observed among the majority of patients in both groups, although the burden of psychological symptoms (SCL-90-R and suicide attempts) was somewhat higher in the VA group.

### Characteristics of involuntarily admitted patients

The substance users included in this study were relatively young, with a mean age of 29 years for IA patients. This is somewhat younger than what was reported by a national study of substance abusers involuntarily admitted to acute psychiatric wards pursuant to the Mental Health Care Act (mean age, 34 years) [12].

In Norway, approximately 70% of persons with SUDs are men [24]. In accordance with this statistic, we observed that, overall, 66% of patients were men. Although the majority of substance users were male, we observed nearly twice as many females among the IA patients compared with the VA patients. Social workers have reported that the reason for the relative excess of female patients in involuntary treatment might be that the service providers considered women more vulnerable and exposed to violence and prostitution [25]. The gender distribution in Norway was not found to differ substantially from what has been found in Sweden [16,26]. Psychiatric hospitals in Norway are characterized by the typical gender distribution, with more women than men among inpatients; however, men are more often involuntarily admitted [8,27]. Overall, 64% of substance users involuntarily admitted to acute psychiatric wards pursuant to the Mental Health Care Act were male [28].

### Severity of drug dependence

IA patients exhibited more severe drug use patterns than VA patients. Significantly more IA than VA patients had been injecting illicit drugs during the last 6 months before admission (71% vs. 46%). Among IA patients, 70% also reported a history of lifetime overdose experiences. According to an overview by Bohnert et al., the lifetime prevalence of overdoses among drug users ranges between 43% and 74% [29]. Hence, this studied population of SUD patients appears to exhibit a particularly high-risk drug use profile.

Polydrug diagnoses tend to be more common among IA patients (*P =* 0.05). According to the EuropASI interviews, IA patients exhibited a consistently higher prevalence of drug use: alcohol, benzodiazepines, other sedatives, and heroin were consumed significantly more often. These findings are similar to those reported in Swedish studies which observed that IA patients were more often drug users and polydrug users, while VA patients more often abused alcohol [16]. The high prevalence of polydrug use is a current trend reported in other studies of psychiatric disorders among SUD patients [12,16]. Therefore, health service providers in countries that are applying involuntary admission pursuant to a social services act should take in consideration these factors. We found that injecting illicit drugs, repeated experience of overdoses and polydrug use were all associated with involuntary hospitalization. Injection of drugs implies both the strongest involvement with drug use and the highest risk of substance-related morbidity and mortality.

### Mental diagnoses and comorbidity

Psychiatric comorbidity has previously been identified among patients admitted involuntarily pursuant to the Mental Health Care Act [11,12,27], including the patients admitted pursuant to the Social Services Act in this study. Although there were no significant differences between the types of mental diagnoses, significantly more IA patients had no mental diagnoses (40% vs. 26%). In an overview of several studies from Sweden, it was observed that 50% to 60% of patients seeking help for substance use disorders did have another psychiatric disorder [16]. Other studies from Norway and other countries have found that among patients seeking help for psychiatric disorders, between 24% and 70% also had a substance abuse problem [30-34]. It has also been shown that comorbidity contributes to re-admission for substance use disorder patients, as well as for those with mental disorders [27,35].

IA patients had significantly more frequent visits to physicians for somatic complaints during the past 6 months (42% of IA patients, compared with 25% of VA patients). This finding indicates that a pattern of severe substance use increases the risk of somatic disorders. High prevalence of chronic disease, acute disease, and injuries among SUD patients have been demonstrated by others [36,37]. This is an often neglected problem that health service providers should take into consideration. SUD patients as demonstrated in this study illuminate characteristics among a vulnerable group of patients that often exhibit combined SUD and mental health problems, but also exhibit somatic disorders in addition. Therefore, comorbidities appear to be particularly relevant. To provide adequate care, treatment centers caring for the needs of these and similar patients would likely benefit from combined expertise in substance use disorders, psychiatric disorders, and somatic disorders [9].

There are some methodological considerations to recognize when interpreting these results. First, the comparison of the two groups, IA patients and VA patients, may be somewhat problematic: VA patients may generally be expected to be more motivated for treatment and more cooperative than IA patients. However, the groups’ characteristics regarding age, education, living conditions, and mental health status were similar. The VA patients had all been considered to qualify for treatment in a hospital. Second, to establish accurate diagnosis for patients with concurrent substance abuse, mental, and somatic disorders can be a challenge. There will always be a risk of misdiagnosing, underreporting, or overreporting illnesses [38].

The main objective of this study was to investigate factors associated with involuntary admission to treatment institutions. Although the social services acts may differ in different countries the characteristics of IA patients found in this study may be similar for patients involuntarily admitted in other countries. We found that 60% of patients IA pursuant to a social services act had comorbid SUD and psychiatric disorder. This comorbidity renders treatment more difficult. Patients diagnosed with comorbidities often require longer treatment duration and more carefully planned care to optimize treatment outcomes. This need presents a major challenge to the health service providers indicating the need to diagnose and treat these patients within a highly competent system. Therefore, clinical routines that better identify and treat SUD and psychiatric disorders among IA patients should be given high priority, as many of the patients would likely benefit from integrated specialist treatment [9].

## Conclusions

This study addresses the knowledge gap and focuses on SUD patients who have been involuntarily admitted to institutions pursuant to a social services act. Our findings of which factors that are associated with involuntary admissions to hospital of these patients may provide useful knowledge that clinical practitioners and authorities in countries using involuntary admissions and treatment of SUD patients would benefit from.

We found that, rather than ICD-10 diagnoses, demographic characteristics and severity of drug use (injecting drugs, overdoses) were associated with involuntary admission to a treatment institution in this study. Women, receiving public welfare benefits, with frequent visits to physicians and drug injection during the past 6 months were more likely to be associated with involuntarily admissions to health services in accordance with the Norwegian Social Services Act of 1993.

In sum, the factors associated with involuntary admission presented in this study indicate that “poverty”, somatic complaints, and a perception of females who are using injection substances as “victims of addiction” characterize patients involuntarily admitted to treatment for substance use disorders. The concurrence of SUD, mental, and somatic complaints presents a major challenge to health service providers also in an international perspective, indicating the need to educate the health providers and to diagnose and treat these patients within a highly competent system.

## Competing interests

The authors declare that they have no competing interests.

## Authors’ contributions

AO has contributed to the study design, in collecting, analyzing and interpreting the data, and prepared the manuscript; ØK has contributed to the study design, in analyzing and interpreting the data, and in preparing of the manuscript; TKL has contributed to the study design and in preparing the manuscript; GS has contributed in collecting the data, and preparing the manuscript; EBAR has contributed in collecting the data, and in preparing the manuscript; AG has contributed to the study design, and in preparing the manuscript; TC has contributed to the study design, in analyzing and interpreting the data, and in preparing the manuscript. All authors have read and approved the final manuscript.

## Pre-publication history

The pre-publication history for this paper can be accessed here:

http://www.biomedcentral.com/1472-6963/13/57/prepub
